# Chemosensory-Related Gene Family Members of the Horn Fly, *Haematobia irritans irritans* (Diptera: Muscidae), Identified by Transcriptome Analysis

**DOI:** 10.3390/insects11110816

**Published:** 2020-11-19

**Authors:** Pia Untalan Olafson, Christopher A. Saski

**Affiliations:** 1 Knipling-Bushland US Livestock Insects Research Laboratory, USDA-ARS, Kerrville, TX 78028, USA; 2 Department of Plant and Environmental Sciences, Clemson University, Clemson, SC 29634, USA; saski@clemson.edu

**Keywords:** horn fly, odorant binding protein, chemosensory binding protein, odorant receptor, gustatory receptor, ionotropic receptor

## Abstract

**Simple Summary:**

Horn flies are blood-feeding ecoparasites that have a significant economic impact on cattle producers in the United States and worldwide. Insecticides have been utilized to reduce horn fly populations, but the development of insecticide resistance has prompted evaluation of alternative control approaches. Compounds isolated from natural products have shown some success in modifying interactions between the horn fly and its host. A more thorough understanding of the horn fly chemosensory pathway would enable identification of species-specific compounds. We assembled a database of genes that are expressed in appendages on the fly head that have a role in sensory input and compared these with genes expressed in adult fly bodies from which heads were removed. We identified genes that were enriched in head appendages and these were similar to previously described genes known to mediate an insect’s response to a chemical stimulus. These included odorant binding proteins and chemosensory binding proteins, as well as receptors that have a role in facilitating responses to odor and/or taste compounds, namely odorant, gustatory, and ionotropic receptors. These findings provide a resource to enable future studies targeting horn fly chemosensation as part of an integrated strategy to control this blood-feeding pest.

**Abstract:**

Horn flies are one of the most significant economic pests of cattle in the United States and worldwide. Chemical control methods have been routinely utilized to reduce populations of this pest, but the steady development of insecticide resistance has prompted evaluation of alternative control strategies. Behavior modifying compounds from natural products have shown some success in impacting horn fly populations, and a more thorough understanding of the horn fly chemosensory system would enable improvements in the development of species-specific compounds. Using an RNA-seq approach, we assembled a transcriptome representing genes expressed in adult female and male horn fly head appendages (antennae, maxillary palps, and proboscides) and adult fly bodies from which heads were removed. Differential gene expression analysis identified chemosensory gene family members that were enriched in head appendage tissues compared with headless bodies. Candidate members included 43 odorant binding proteins (OBP) and 5 chemosensory binding proteins (CSP), as well as 44 odorant receptors (OR), 27 gustatory receptors (GR), and 34 ionotropic receptors (IR). Sex-biased expression of these genes was not observed. These findings provide a resource to enable future studies targeting horn fly chemosensation as part of an integrated strategy to control this blood-feeding pest.

## 1. Introduction

Horn flies (*Haematobia irritans irritans* (L.)) are obligate ectoparasites of pastured cattle that blood-feed almost hourly over a 24 h period and, as such, are somewhat permanently associated with their host. Management of these fly populations has relied primarily on topical application of synthetic insecticides delivered via ear tags, boluses, and dust bags, as well as on-animal pour-ons and sprays [1]. While effective, insecticide resistance development in horn flies is well documented [2] and use of management practices to minimize the impact of fly resistant populations on pastured cattle has been challenging [3,4]. A need for pest management tools in organic livestock production settings and a desire to reduce the ecological impact of broad spectrum insecticides have increased interest in the development of nonchemical control strategies, including the potential for a vaccine targeting horn flies [5,6] and mechanical approaches for the removal of flies from bovine hosts [7]. Encouraging results have also been obtained using natural compounds to manipulate horn fly behavior. The repellent effect of geranium, lemongrass, peppermint, catnip, geraniol, and coconut oil constituents have been documented against horn flies in a laboratory setting [8,9,10,11]. Further, on-animal, field evaluation of the repellency of medium chain length fatty acid derivatives and 2% geraniol against the horn fly suggested these compounds impacted the mating status of on-animal populations and were effective at reducing blood-feeding by female horn flies and at repelling older females [12,13,14]. While the duration of activity is currently limited and the desired response can require large quantities of compound, the results are promising. Designing behavior modifying compounds that are more species-specific would also be beneficial, and this can be achieved by targeting chemosensory pathways to identify molecules activated by these compounds (i.e., repellents and attractants). This intriguing model has been proposed for managing various insect pests of human and animal health importance [15,16,17,18].

The mating behavior of the horn fly has been described in both a laboratory and field setting. Mating occurs on-animal, and the orientation of males to females prior to abdominal tapping may involve semiochemical cues [19,20]. Using biological assays, Bolton et al. [21] observed horn fly male attraction to female cuticular hydrocarbon extracts and, upon fractionation, identified monoolefins as the primary compounds eliciting the male attraction response. It is unclear whether the horn fly response was mediated by gustatory perception of the cuticular hydrocarbons or olfactory perception of volatile compounds resulting from breakdown of the cuticular hydrocarbons. Gravid horn fly females intermittently leave their host to oviposit in freshly laid bovine manure within five minutes of fecal deposition [22,23]. Horn fly oviposition involves the integration of mechanosensory cues to monitor host behaviors associated with fecal deposition, as well as possibly olfactory and contact chemosensory reception by flies to orient to the pat and evaluate it for suitability, e.g., moisture content [23]. Birkett et al. [24] identified host-emitted semiochemicals that elicit an antennal response in horn flies, indicating a role for olfaction in host localization.

**Figure 1:**
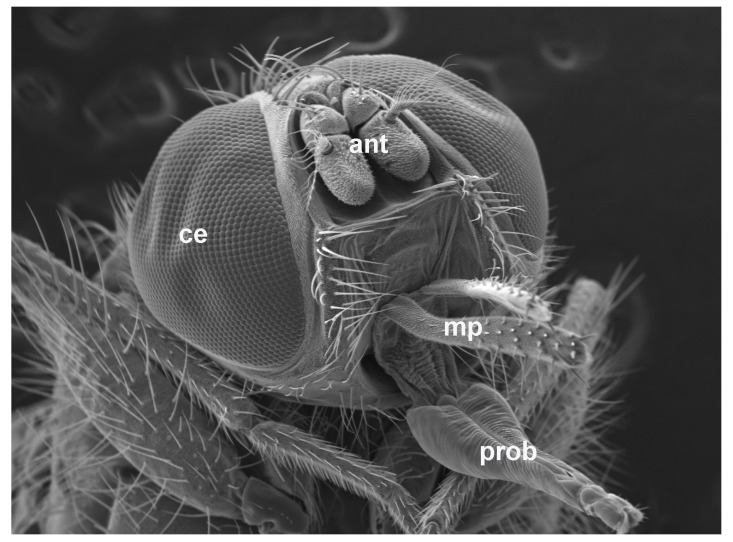
Scanning electron micrograph of a male horn fly head. Antennae (ant), maxillary palps (mp), and the proboscis (prob) were dissected as part of this study. Antennae are located between the compound eyes (ce), while the maxillary palps are located at the base of the proboscis.

The distribution of sensilla on horn fly antennae, mouthparts, and the ovipositor has been described using scanning electron microscopy [25,26,27,28]; however, genes regulating horn fly chemosensory pathways are understudied [29]. While Domingues et al. [30] published a horn fly transcriptome representing genes expressed in whole adults that survived exposure to permethrin, a pyrethroid insecticide, and permethrin plus a synergist versus untreated adults, we aimed to target genes expressed in tissues likely enriched in chemosensory gene families. To that end, we employed RNA-seq analysis to compare gene expression in horn fly head appendages (antennae, maxillary palps, and the proboscis; Figure 1) from blood-fed, mated female and male adults and the headless bodies of these females and males. Using this transcriptome, we identified candidate horn fly chemosensory gene families enriched in head appendages.

## 2. Materials and Methods

### 2.1. Horn Fly Tissue Collection and Total RNA Isolation

Tissues for this study were dissected from specimens of a colonized strain of horn flies maintained by the Knipling–Bushland US Livestock Insects Research Laboratory (Kerrville, TX, USA) at 27 °C, 60% relative humidity, and a photoperiod of 12 h light:12 h dark. Adult horn flies were sustained on a supply of citrated bovine blood offered with a saturated feminine napkin, and they were aspirated from the rearing cage at 7 d post-emergence. At this stage, horn flies have already mated and females are beginning to oviposit. Adult flies were chill anesthetized to sort females and males, and these were snap frozen in liquid nitrogen for dissection of head appendages and for removal of heads to obtain thorax-abdomen (headless bodies) material. Legs and wings were not removed from the headless bodies.

Head appendages (antennae, maxillary palps, and proboscides) of female or male flies were dissected on dry ice from the frozen adult specimens (*n* = 100 per appendage per sex, pooled). As the organs were dissected, they were immediately placed in TRI Reagent^®^, macerated with a disposable DNAse/RNase-free Kontes^®^ Pellet Pestle^®^ (DWK Life Sciences (Kimble), Millville, NJ, USA), and stored at −80 °C until processed. The head of adult female or male horn flies was removed from *n* = 20 specimens per replicate, resulting in "headless body" material. These were processed in the same manner as the head appendages. Total RNA was isolated from these samples using the Zymo Direct-zol™ method (Zymo Research, Irvine, CA, USA) with on-column DNAse treatment (TURBO™ DNase, ThermoFisher Scientific, Waltham, MA, USA).

### 2.2. Library Preparation, Sequencing, and Pre-Processing

Total RNA was assessed for integrity with an Agilent Bioanalyzer 2100 (Agilent Technologies, Santa Clara, CA, USA) and assigned an RNA integrity number (RIN). Each sample had at least a RIN of 7.0. Total RNA was quantified with the RNA HS Assay Kit (ThermoFisher Scientific) on a Qubit 2.0 fluorometer (ThermoFisher Scientific) and normalized to a total RNA mass of 1 μg for each sample. Strand-specific mRNA seq libraries were prepared for each sample with the TruSeq Stranded Total RNA kit (Illumina, San Diego, CA, USA) following the manufacturers recommended procedures. The resulting libraries were quantified using a Qubit 2.0 fluorometer (ThermoFisher Scientific) and quantitative PCR. Size distribution was analyzed using an Agilent Bioanalyzer 2100 (Agilent Technologies). Qualified libraries were sequenced, and sequencing was collected using 250 cycles (125 bp × 2) on an Illumina HiSeq2500. Raw sequence reads were assessed for quality with the fastqc software tool (https://www.bioinformatics.babraham.ac.uk/projects/fastqc/). Sample files were trimmed of low-quality bases and contaminating adapter sequences with the Trimmomatic software package [31]. The data has been deposited at GenBank under the accession SAMN16338536 and BioProject PRJNA666941.

### 2.3. De Novo Transcriptome Assembly, Annotation, and Functional Categorization

Trimmed read pairs from all samples were combined manually and approximately 120 million read pairs (approx. 30 billion bases) were used as input for a global de novo transcriptome assembly with the Trinity software package [32], v.2.8.2. This base assembly was further screened for transcripts that contained internal stop codons or unlikely coding sequences with the TransDecoder plugin of the Trinity software package [32]. Transcripts were collapsed into the longest predicted unigene with the cdHit software [33] using a 98% identity threshold. Unigene sequences were annotated for homology with the top blast-hit to the SwisProt reference protein database [34] and for function using InterProScan v5.46-81.0 [35] with analysis that included: Pfam (33.1) [36] and PANTHER (14.1) [37]. Gene ontology terms were assigned using the -goterm parameter when running InterProScan. Subsets of sequences were evaluated for presence of signal peptides and transmembrane domains using the SignalP-5.0 and TMHMM v. 2.0 web servers, respectively [38,39].

### 2.4. Differential Gene Expression and Gene Ontology Enrichment Analyses

Transcript quantification was performed by first aligning strand-specific read pairs (an average of 30 million read pairs for each individual sample) to the reference unigene set with the Bowtie2 short-read aligner v.2.3.4.1 [40], and abundance estimates (trimmed mean of M-values (TMM), transcript per million (TPM), fragments per kilobase per million mapped (FPKM) reads) were determined with RNA-Seq by Expectation-Maximization (RSEM v.1.3.3) [41]. Differentially expressed transcripts were determined with edgeR v3.14.0 [42] in the R statistical computing language and environment v.4.0.2. Stringent criteria were used to identify differentially expressed transcripts. Significant differences were based on an adjusted *p* < 0.01 calculated using the Benjamini–Hochberg false discovery rate (FDR) method with an FDR of 0.05. To visualize differential expression, genes were partitioned into expression clusters by manually creating an expression matrix of genes whose log_2_ normalized and centered FPKM+1 values were differentially expressed (log Fold Change (FC)) ≥ 2 and *p*-value ≤ 0.001. Hierarchal clustering of genes was performed with the fastcluster R software package and cut at 60% max height of the tree with custom scripts. Gene Ontology (GO) enrichment analysis was performed with either the clusters produced as described above or with individual RNA-seq datasets (female or male, head appendage or headless bodies) as input to the GoSeq R software package [43]. Heatmaps were generated in R v.4.0.2 with the heatmap.2 function.

### 2.5. Phylogenetic Analysis

Chemosensory gene family sequences from *Stomoxys calcitrans*, *Musca domestica*, and *Drosophila melanogaster* were obtained from Uniprot and Olafson et al. [44]. Sequences were used in tBLASTN searches to identify orthologs in the horn fly transcriptome assembled as part of this study. Amino acid sequences from each family were aligned with the MUSCLE algorithm [45], and the alignments trimmed with the trimAl tool using the “–strictplus” option [46]. The trimmed alignment was used to construct a maximum likelihood phylogeny with the web server version of IQ-TREE software [47] using the best-fit substitution model and branch support assessed with 1000 replicates of UFBoot bootstrap approximation [48]. Odorant binding protein domains of dimers were separated for phylogenetic analysis and labeled “a” and “b”, and the tree was rooted at midpoint. The phylogenetic tree of odorant receptors was rooted with the highly conserved odorant co-receptor, ORCO, as the outgroup, while the tree of gustatory receptors was rooted with the carbon dioxide receptor subfamily as the outgroup. The insect ionotropic receptor tree was rooted with co-receptors Ir8a/Ir25a as the outgroup.

## 3. Results

### 3.1. Transcriptome Sequencing, Assembly, and Differential Expression Analysis

Raw reads from four conditions were separately trimmed then assembled de novo using the Trinity algorithm. The four conditions consisted of 7 d fed, mated male or female head appendages (antennae, maxillary palp, and proboscis) and 7 d fed, mated male or female headless bodies. Total numbers of read pairs for each condition are summarized in Table 1. The resulting assembly comprised 23,806 unigenes, labeled HF_Trans_#, with an averag length of 1137 bp. Throughout the manuscript, these transcripts are referred to as Hirr_#. The single replicate RNA-seq datasets were mapped to the de novo assembly and analysed to identify genes signficantly differentially expressed between females and males, as well as between pooled head appendages and headless bodies. While only one replicate sample per condition was analyzed, biological variation was partly accounted for by isolating total RNAs after pooling tissues from 100 individuals per sex for the head appendage datasets and pooling 20 individuals per sex for the headless body datasets. Complete output from these analyses can be found in Supplementary File 1. Differentially expressed genes were defined as those that had −2 ≥ logFC ≥ 2. Using this criteria, there were 2449 differentially expressed transcripts in the “female head appendage” versus “female headless bodies” comparison; 2752 differentially expressed transcripts in the “male head appendage” versus “male headless bodies” comparison; and lastly, 593 differentially expressed transcripts in the “female head appendage” versus “male head appendage” comparison.

**Figure 2:**
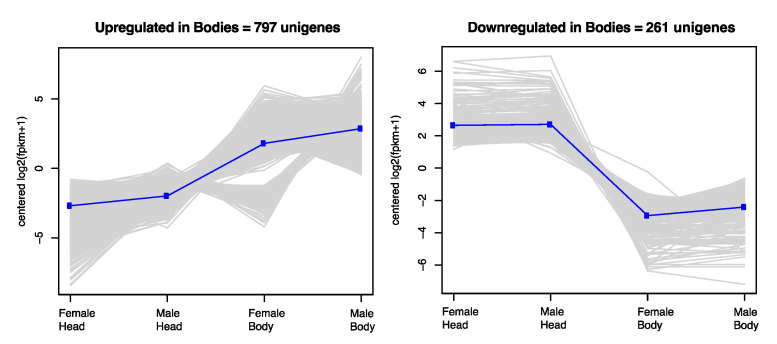
Hierarchical clustering of the differentially expressed genes from the head appendage (Head) and headless body (Body) datasets. Differentially expressed transcripts were partitioned into two clusters representing genes upregulated and downregulated in headless bodies (Supplementary File 2).

Differentially expressed transcripts were partitioned into two clusters representing genes upregulated and downregulated in headless bodies (Figure 2). Gene ontology analysis of clusters representing transcripts upregulated in headless bodies identified significant enrichment of categories associated with serine-type endopeptidase activity, proteolysis, chitin binding, carbohydrate metabolic process, transmembrane transporter activity, phospholipase A1 activity, sperm chromatin condensation, metallopeptidase activity, spermatogenesis, and metallocarboxypeptidase activity (Supplementary File 2). In contrast, gene ontology analysis of clusters representing transcripts downregulated in headless bodies identified significant enrichment of categories associated with odorant binding, olfactory receptor activity, sensory perception of smell, membrane, and ligand-gated ion channel activity in those (Supplementary File 2).

### 3.2. Enriched Gene Ontology Categories of Differentially Expressed and Tissue-Specific Trancripts

Subsequent gene ontology analysis of differentially expressed transcripts between each of the datasets identified enrichment of functional categories (Supplementary File 3). The most highly significant groups enriched in head appendages included odorant binding, olfactory receptor activity, ligand-gated ion channel activity, heme binding, ionotropic glutamate receptor activity, DNA-binding transcription factor activity, oxidoreductase activity, and iron ion binding. Further analysis of transcripts that were specific to the male head appendage identified significant enrichment in ATP binding, catalytic activity, protein kinase activity, protein phosphorylation, and oxidation-reduction processes categories, while significantly enriched categories in female-specific head appendage transcripts were nucleic acid binding and regulation of gene silencing by miRNAs.

### 3.3. Candidate Horn Fly Chemosensory Gene Family Members Identified from Transcriptome Analysis of Pooled Head Appendages and Headless Bodies

Annotated chemosensory gene family members described from closely related muscids (Musca domestica and Stomoxys calcitrans) and D. melanogaster were used to identify orthologous sequences from the horn fly transcriptome assembled as part of this study. This query identified members of non-receptor carrier protein gene families, as well as seven transmembrane and glutatmate ionotropic chemoreceptor gene families. A summary of statistically supported, differentially expressed chemosensory genes is available as Supplementary File 4. Only those transcripts that were −2 ≥ logFC ≥ 2 were considered in reporting differential expression.

#### 3.3.1. Horn Fly Odorant Binding Proteins (OBP) and Chemosensory Proteins (CSP)

**Figure 3:**
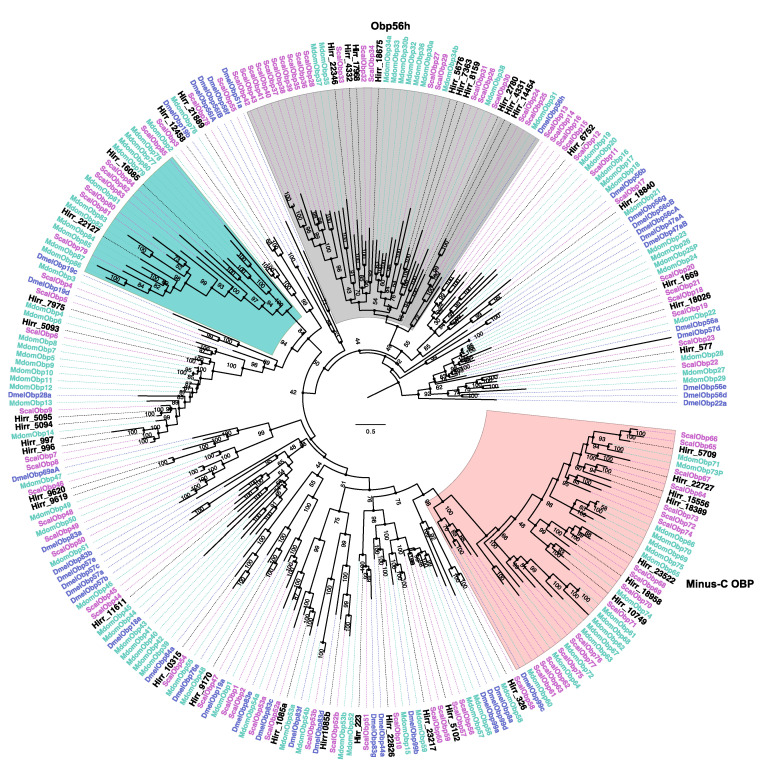
Phylogenetic relationship of *Haematobia irritans* candidate odorant binding proteins (OBP) with those of *Stomoxys calcitrans*, *Musca domestica,* and *Drosophila melanogaster*. The *H. irritans* (Hirr) genes are labeled in black, while the *S. calcitrans* (Scal) and *M. domestica* (Mdom) genes are labeled in pink and teal, respectively. *D. melanogaster* (Dmel) genes are labeled in purple. The Minus-C OBP lineage is shaded in pink, while the lineage with expansions relative to DmelObp56 h is shaded in grey. A lineage of muscid OBPs with no apparent orthologue in *Drosophila* is shaded in teal.

**Figure 4:**
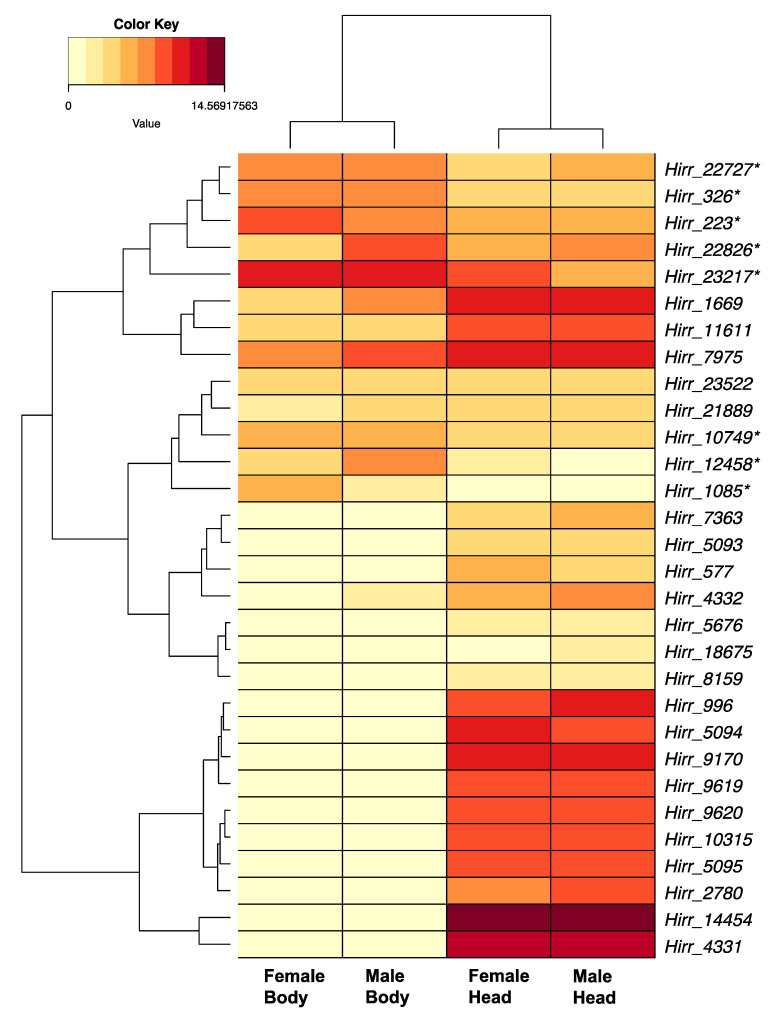
Heat map and hierarchical clustering dendrogram of *Haematobia irritans* candidate OBP transcripts in female and male head appendages (Head) and headless bodies (Body). Log-transformed TPM values were used to construct the heat map. OBPs expressed significantly higher in bodies versus head appendages are identified by an asterisk (*). Thirteen candidate OBP transcripts are not depicted, as they had relatively low expression values. Dendrogram reflects transcripts with similar expression patterns, identified by hierarchical clustering applied to expression values (rows) and dataset categories (columns).

A total of 43 horn fly transcripts encoded an OBP domain, which is typically characterized by six highly conserved cysteine residues. Twelve of the sequences were partial, ranging in size from 104 to 126 amino acid residues and missing either the 5′ or 3′ end. All full-length sequences encoded a signal peptide motif suggesting secretion. Of these transcripts, 34 belonged to the Classical OBP subclass with the six cysteine residues exhibiting a C_1_–X_26–34_–C_2_–X_3_–C_3_–X_25–38_–C_4_–X_8–11_–C_5_–X_8_–C_6_ pattern. One transcript (Hirr_1085) belonged to the dimer OBP subclass with two classical OBP motifs fused, and eight transcripts belonged to the Minus-C subclass. The horn fly Minus-C OBPs encoded five cysteine residues, lacking the cysteines at the classic C_2_ and C_5_ positions; however, seven of these transcripts encoded an alternate cysteine located seven residues upstream of the C_4_ position and one (Hirr_326) encoded an alternate cysteine three residues downstream of the C_5_ position (Figure S1). A phylogenetic comparison of these OBPs with orthologues from closely related muscids and D. melanogster is presented in Figure 3. The horn fly Minus-C OBPs cluster with 17 S. calcitrans OBPs that have a similar cysteine arrangement. Of these 43 OBP transcripts, 20 had 2- to 13-fold significantly higher expression in head appendages versus headless bodies, and a single transcript (Hirr_23217) was expressed 3.9-fold higher in female versus male head appendage datasets (Figure 4; Table 2). Conversely, eight of the transcripts were expressed significantly higher in headless bodies versus head appendages, one of which was Hirr_23217 (Figure 4).

**Figure 5:**
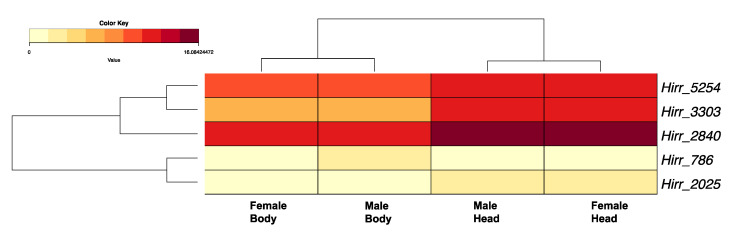
Heat map and hierarchical clustering dendrogram of *Haematobia irritans* candidate chemosensory protein (CSP) transcripts in female and male head appendages (Head) and headless bodies (Body). Log-transformed TPM values were used to construct the heat map. Dendrogram reflects transcripts with similar expression patterns, identified by hierarchical clustering applied to expression values (rows) and dataset categories (columns).

Five transcripts (Hirr_786, Hirr_2025, Hirr_2840, Hirr_3303, Hirr_5254) encoded a CSP motif characterized by four highly conserved cysteine residues, all in a C_1_–X_6_–C_2_–X_18_–C_3_–X_2_–C_4_ pattern. Four of the transcripts encoded a signal peptide, suggesting secretion (Hirr_3303 did not). Three of the transcripts exhibited 3- to 5- fold higher expression in head appendage versus headless bodies, while one transcript (Hirr_786) was expressed two-fold higher in male headless bodies than male head appendages (Figure 5).

#### 3.3.2. Horn Fly Odorant Receptors (OR)

**Figure 6:**
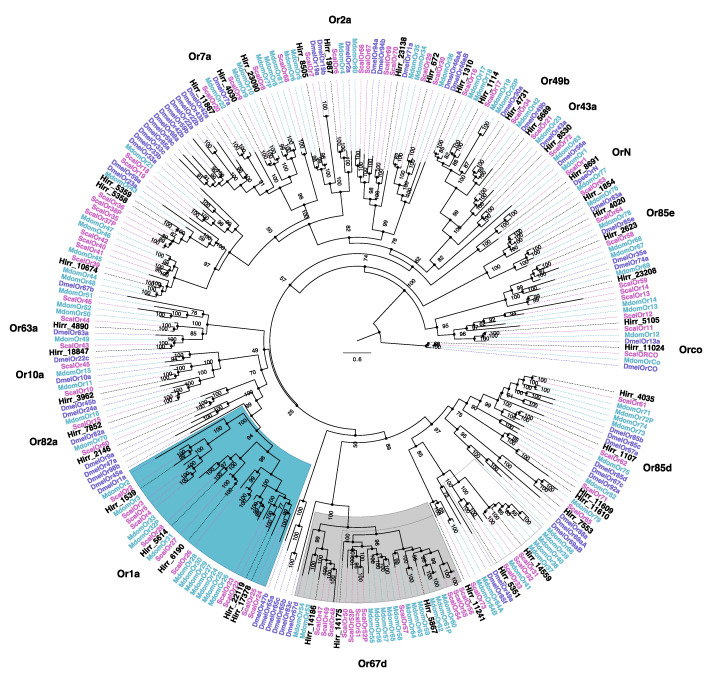
Phylogenetic relationship of *Haematobia irritans* candidate odorant receptors (OR) with those of *Stomoxys calcitrans*, *Musca domestica,* and *Drosophila melanogaster*. The *H. irritans* (Hirr) genes are labeled in black, while the *S. calcitrans* (Scal) and *M. domestica* (Mdom) genes are labeled in pink and teal, respectively. *D. melanogaster* (Dmel) genes are labeled in purple. Lineages that include apparent expansions in muscids relative to DmelOr67d and DmelOr1a are shaded in grey and teal, respectively. Individual *Drosophila* genes for which 1:1 horn fly candidate orthologues were identified are labeled on the edge to assist with finding them in the tree.

**Figure 7:**
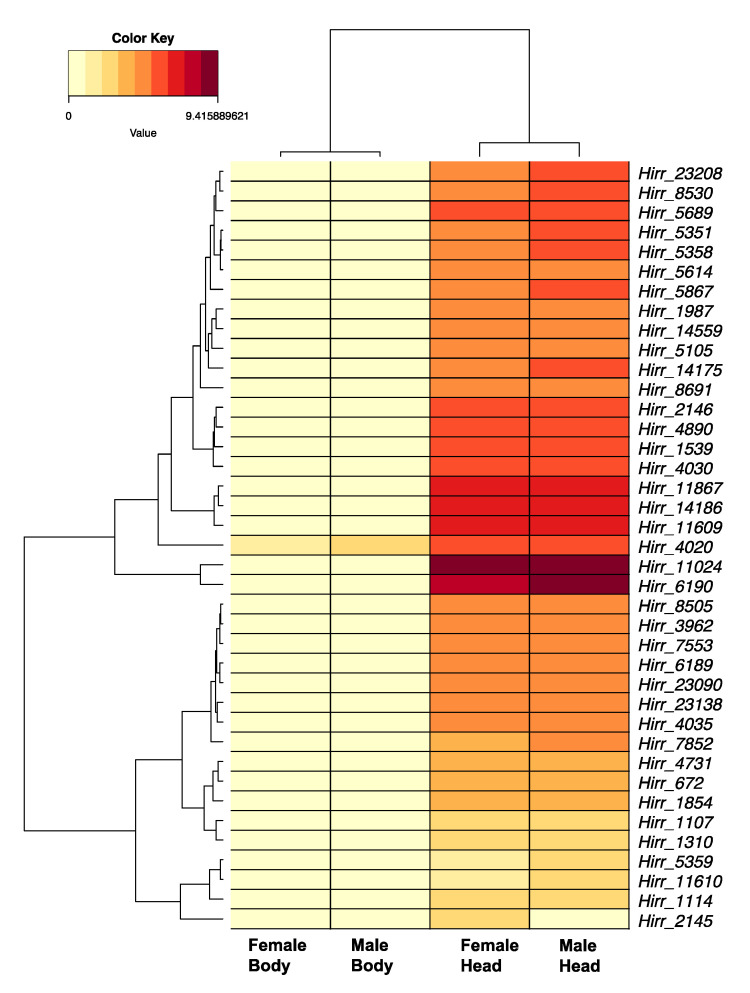
Heat map and hierarchical clustering dendrogram of *Haematobia irritans* candidate OR transcripts in female and male head appendages (Head) and headless bodies (Body). Log-transformed TPM values were used to construct the heat map. Five candidate OR transcripts are not depicted, as they had low levels of expression. Dendrogram reflects transcripts with similar expression patterns, identified by hierarchical clustering applied to expression values (rows) and dataset categories (columns).

A total of 44 horn fly transcripts encoded a conserved OR domain, seven transcripts of which were not full-length (109–305 amino acids). The full-length transcripts encoded 4–7 transmembrane domains based on TMHMM analysis, while those that were truncated encoded between 1 and 4 transmembrane domains. A phylogenetic comparison of these ORs with orthologues from closely related muscids and D. melanogaster is presented in Figure 6. An orthologue of the highly conserved co-receptor Orco was detected (Hirr_11024), as well as an orthologue of the D. pseudobscura OrN, Hirr_8691. Additional 1:1 orthologs with D. melanogaster ORs included: Hirr_4020 (DmelOr85e), Hirr_5689 (DmelOr43a), Hirr_4731 (DmelOr49b), Hirr_1987 (DmelOr2a), Hirr_18847 (DmelOr63a), Hirr_3962 (DmelOr10a), Hirr_2146 (DmelOr82a), and Hirr_1107 (DmelOr85d). OR transcript expression was detected predominantly in head appendage tissues (Figure 7; Supplementary File 4), and if detected in headless bodies the TPM values were quite low. One exception was Hirr_4020, which was expressed in all datasets.

#### 3.3.3. Horn Fly Gustatory Receptors (GR)

**Figure 8:**
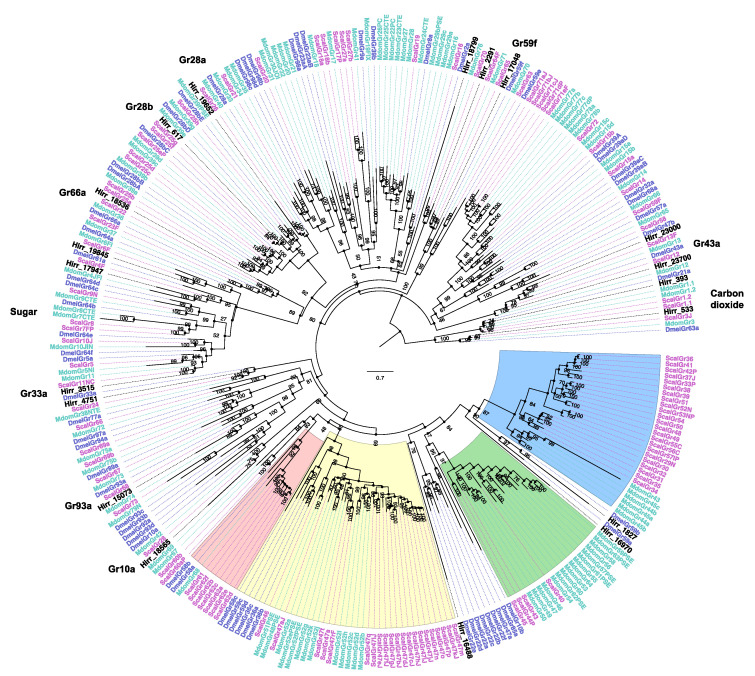
Phylogenetic relationship of *Haematobia irritans* candidate gustatory receptors (GR) with those of *Stomoxys calcitrans*, *Musca domestica,* and *Drosophila melanogaster*. The *H. irritans* (Hirr) genes are labeled in black, while the *S. calcitrans* (Scal) and *M. domestica* (Mdom) genes are labeled in pink and teal, respectively. *D. melanogaster* (Dmel) genes are labeled in purple. Shaded regions identify four different bitter taste receptor clades that are expanded in *S. calcitrans* and *M. domestica*, as described in [44]. The color pattern of clades used in [44] is reflected here. Individual *Drosophila* genes are labeled on the edge to assist with finding them in the tree.

**Figure 9:**
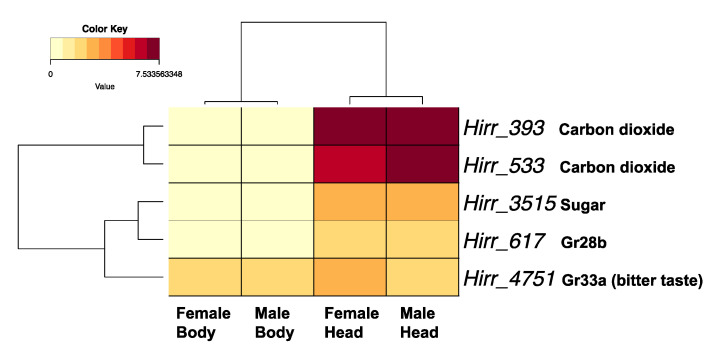
Heat map and hierarchical clustering dendrogram of *Haematobia irritans* candidate GR transcripts in female and male head appendages (Head) and headless bodies (Body). Log-transformed TPM values were used to construct the heat map. These five represent the most abundantly expressed transcripts encoding GRs. Dendrogram reflects transcripts with similar expression patterns, identified by hierarchical clustering applied to expression values (rows) and dataset categories (columns).

A chemosensory or trehalose receptor domain, indicative of GRs, was detected in 27 horn fly transcripts. However, the majority were not full-length and the proteins they encoded ranged in size from 99 to 511 amino acids (average: 232 ± 27 amino acids). Of these 27, four did not encode a transmembrane domain based on TMHMM analysis and were not included in phylogenetic comparisons. Further, while proteins encoded by another four transcripts had at least one transmembrane domain (Hirr_22667 (6 TM), Hirr_22843 (4 TM), Hirr_21410 (1 TM), and Hirr_18054 (1 TM)), they were also excluded from the final phylogenetic comparison, as they were represented by very long branches for reasons that are not apparent. The relationship of the horn fly GRs with orthologues from closely related muscids and D. melanogaster is presented in Figure 8. Hirr_393 and Hirr_533 are orthologues of the carbon dioxide receptors, and they had the highest expression levels among these candidate GR transcripts with significantly higher expression in head appendages versus headless bodies (Figure 9). Three transcripts are orthologous to sugar receptors, two of which were detected in head appendage datasets (Hirr_19845 and Hirr_3515) and one of which was detected only in the male headless bodies dataset (Hirr_17947). Hirr_3515 was expressed significantly higher in head appendages versus headless bodies (Supplementary File 4).

#### 3.3.4. Horn Fly Ionotropic Receptors (IR)

**Figure 10:**
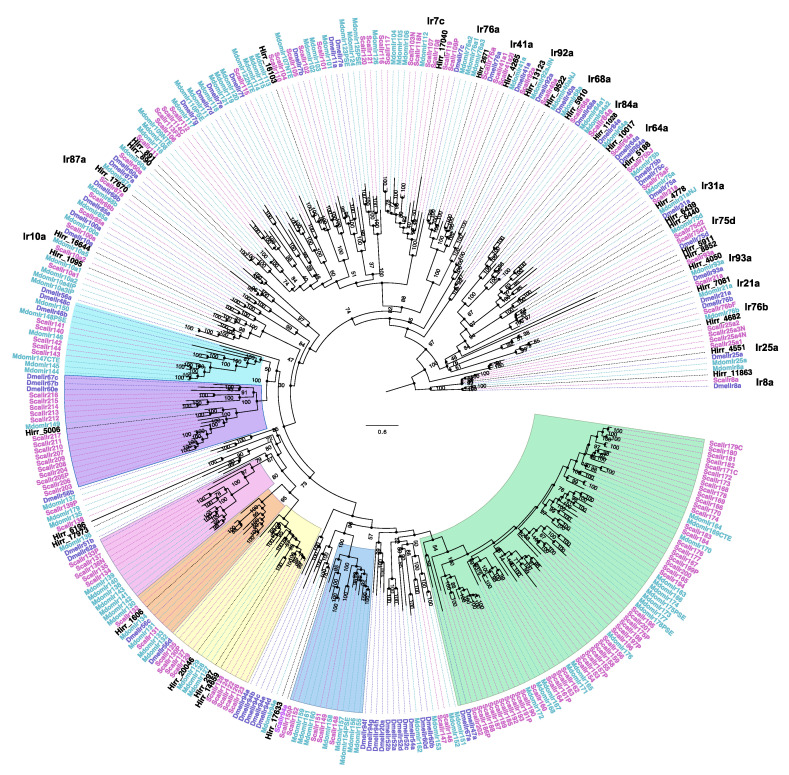
Phylogenetic relationship of *Haematobia irritans* candidate ionotropic receptors (IR) with those of *Stomoxys calcitrans*, *Musca domestica,* and *Drosophila melanogaster*. The *H. irritans* (Hirr) genes are labeled in black, while the *S. calcitrans* (Scal) and *M. domestica* (Mdom) genes are labeled in pink and teal, respectively. *D. melanogaster* (Dmel) genes are labeled in purple. Shaded regions identify genes in the Ir20a clade that are expanded in *S. calcitrans* and *M. domestica*, as described in [44]. Color pattern of clades used in [44] is reflected here. Individual *Drosophila* genes are labeled on the edge to assist with finding them in the tree.

A total of 34 horn fly transcripts encoded a conserved ionotropic glutamate receptor domain and were annotated as ionotropic receptors using the PANTHER database. A phylogenetic comparison of these sequences with IR proteins from closely related muscids and D. melanogaster (Figure 10) identified the horn fly orthologues of IR8a (Hirr_11863) and IR25a (Hirr_4551), which are co-receptors that form heterodimers with tuning IRs to mediate a sensory response [49]. One transcript (Hirr_11863) was excluded from the phylogeny, as it was represented by a long branch. The 34 transcripts are predominantly expressed in the head appendage tissues relative to the headless bodies. One transcript (Hirr_1095) was expressed 2.7-fold higher in male versus female headless bodies (Supplementary File 4).

## 4. Discussion

The description of chemosensory gene families from muscid pests of livestock has been limited to the stable fly and the house fly [44,50]. Here, we assembled a horn fly transcriptome representing genes expressed by fed, mated, adults, and we identified genes that were expressed at a higher level in head appendage tissues versus headless bodies. Because our head appendage datasets represented a pool of antennae, maxillary palps, and proboscides, tissue-specific comparisons could not be made. Regardless, this approach enabled us to describe head appendage transcripts enriched for genes related to sensory perception and to identify candidate members of chemosensory-related gene families from the horn fly.

OBPs and CSPs are small (10–30 kDa), globular proteins that can be found in the lymph surrounding sensilla in the insect antenna, and they bind a variety of ligands including odorant compounds [51,52]. They are characterized by a signal peptide directing secretion and a conserved arrangement of cysteine residues that form disulfide bonds, with OBPs and CSPs having a signature of six and four cysteines, respectively [53,54]. This difference in number of disulfide bonds formed is reflected in the crystal structures for each of these proteins [55,56]. We identified horn fly candidates for 43 OBPs and 5 CSPs, and our data supported enriched expression of these genes in head appendages of both females and males versus headless bodies (Table 1, Supplementary File 4). We also identified at least eight OBPs that are expressed at a 2- to 8-fold higher level in headless bodies versus head appendages (Figure 4) and one CSP with a similar 3-fold higher level of expression (Figure 5). Detection of these genes in non-olfactory tissues has been previously reported [57,58], further supporting their extended role as carrier proteins with functions beyond olfaction. Pitts et al. [59] observed a comparable enrichment of OBPs in transcriptomes of female or male *Anopheles gambiae* antennae and maxillary palps, as well as several OBPs enriched in whole bodies. Further, OBPs were the most robustly expressed chemosensory genes in a Drosophila antennal-specific transcriptome with evidence for expression in non-chemosensory tissues [60]. Phylogenetic comparison of the 43 horn fly OBPs to those from *S. calcitrans*, *M. domestica*, and *D. melanogaster* identified at least eight simple 1:1:1 orthologous relationships (Figure 3). Expansions in muscid OBP gene families relative to *D. melanogaster* have been reported as part of genome sequencing analyses [44,50], and this is further supported by our data from horn flies. Examples of this are evident in the *D. melanogaster* Obp56h lineage and the lineage comprising the Minus-C OBPs. Gene silencing of *Drosophila* Obp56h resulted in a modified cuticular hydrocarbon profile of male *Drosophila* and in the reduction of 5-T, a hydrocarbon that is produced by males and thought to delay onset of courtship [61], thus it is believed to have a role in male mating behavior. Ten *H. irritans* OBPs, along with the previously reported 20 *S. calcitrans* and 9 *M. domestica* OBPs, reside on the same lineage as DmelObp56h and further supports duplication of this OBP in the muscids (Figure 3). Interestingly, two OBPs from this lineage, *Hirr_14454* and *Hirr_4331*, have the highest level of OBP expression detected from female or male head appendages. Another example of expansion in this gene family was found in the Minus-C OBP lineage, relative to *D. melanogaster* Obp99c ([62]; Figure 3). Seven *H. irritans* OBPs form part of this expansion along with 17 *S. calcitrans* and 16 *M. domestica* OBPs that share the loss of the conserved cysteines at the C_2_ and C_5_ positions with an alternate cysteine located upstream of C_4_. Two of these transcripts, *Hirr_22727* and *Hirr_10749*, have significantly higher expression in headless bodies than head appendages suggesting a non-olfactory role (Table 1). Another member of this lineage, *Hirr_326*, lost the conserved C_2_ and C_5_ positions but had an alternate cysteine downstream to the C_5_ residue. This transcript was also expressed at a higher level in headless bodies versus head appendages (Table 1). While there is evidence that Minus-C OBPs bind odor compounds [63], their association with behavior is unknown.

An arthropod’s perception of environmental cues is mediated by three major chemosensory receptor gene families: odorant, gustatory, and ionotropic [64,65]. Insect odorant receptors are ion channels characterized by a seven transmembrane domain, and odor compounds are perceived by heterodimerization of a highly conserved odorant co-receptor, ORCO, and a ligand binding odorant receptor [66]. Phylogenetic comparison of the 44 candidate *H. irritans* ORs with those annotated from *S. calcitrans*, *M. domestica*, and *D. melanogaster* identified 15 genes with a simple 1:1:1 orthologous relationship. This included the most abundantly expressed OR in this study, *Hirr_11024*, which is an orthologue of ORCO that was previously described from the horn fly [29]. Another, *Hirr_8691*, is an orthologue of the *D. pseudobscura* OrN, which was lost from *D. melanogaster* [67]. To be consistent with the naming system in other muscid flies, this OR will be identified as *Hirr*Or1. The remainder of the horn fly ORs cluster with muscid genes that are duplicated relative to *D. melanogaster*. An example of this is the lineage that includes a single *D. melanogaster* Or67d gene compared with three *H. irritans*, 11 *S. calcitrans* and 13 *M. domestica* OR genes. In *Drosophila*, DmelOr67d has a role in recognizing a male-specific mating pheromone, cis-vaccenyl acetate [68], that regulates mating behaviors [69]. Its role in muscids, which exhibit different mating behaviors, is unknown. Another example is the lineage comprising a single *D. melanogaster* Or1a with six *H. irritans*, 11 *S. calcitrans*, and 11 *M. domestica* OR genes. In *Drosophila*, DmelOr1a is larval-specific receptor [70] that is responsive to 2-hexenal and acts in concert with other larval ORs to mediate responses to odors [71]. Life-stage specific expression of the horn fly transcripts has yet to be determined. Given the enrichment of olfactory sensory neurons on antennae, it was not unexpected that the OR genes were predominantly expressed in head appendage tissues with low TPM values in headless bodies, if detected. Interestingly, *Hirr_4020*, an orthologue of *D. melanogaster* Or85e, was detected at a relatively high level in female and male headless bodies when compared with detection of the other ORs from bodies. In *D. melanogaster*, Or85e is co-expressed with DmelOr33c in the palp basiconic 2 sensillum of the maxillary palp, and it is highly responsive to the monoterpenoid fenchone [72,73]. Olafson [29] reported detection of *Orco* in horn fly maxillary palps, proboscis, and ovipositor, suggesting there is a ligand-binding OR expressed in these tissues; however, Fernandes et al. [27] report no olfactory sensilla upon SEM analysis of the horn fly maxillary palps. This database of horn fly ORs can be used to systematically identify whether ligand-binding ORs are expressed in these tissues, as well as identify others that are important to different horn fly physiological states and lifestages.

Insect gustatory receptors are expressed in taste sensilla that can be found on the labellum, tarsi, and wing margins (reviewed in [65]). Phylogenetic analysis of the 27 candidate horn fly GRs identified orthologues of the highly conserved carbon dioxide receptors Gr21a (*Hirr_393*) and Gr63a (*Hirr_533*) [74]. These were the most abundantly expressed GR transcripts in head appendages and were significantly enriched in these tissues versus headless bodies. Horn flies require a bloodmeal for survival and egg development, but they can be sustained on sucrose in the laboratory for several days if needed. Orthologues were identified to three *Drosophila* sugar receptors, DmelGr61a (*Hirr_17947*), DmelGr64a (*Hirr_19845*), and DmelGr64f (*Hirr_3515*) [75,76,77], one of which (*Hirr_3515*) was in the top five most abundant GR transcripts in head appendages. Further, orthologues of DmelGr43a, *Hirr_23000* and *Hirr_23700*, were identified with duplications evident in muscid flies relative to *Drosophila* (Figure 8). Sato et al. [78] defined the narrowly tuned response of the DmelGr43a receptor to fructose, and Miyamoto et al. [79] characterized its role as a sensor for fructose levels in the hemolymph, transmitting nutrient status to the brain. Horn flies orient to their host fairly quickly after emergence and are less likely to rely on alternative energy sources; further studies are required to identify the role of sweet receptors in horn fly biology. Genomic analysis of *S. calcitrans* and *M. domestica* identified an expansion in GRs that have a likely role in bitter taste perception (Figure 8; red, yellow, green, and blue shaded regions). None of the horn fly candidate GRs from this study clustered with these expanded lineages, and this could be a reflection of the reduced interaction that horn flies have with the landscape given the almost permanent relationship with their bovine host. Orthologues to *Drosophila* bitter taste receptors DmelGr66a (*Hirr_18536*) and DmelGr33a (*Hirr_4751*) [80,81] were identified, and *Hirr_4751* was detected in both head appendages and headless bodies (Figure 9). *Hirr_617*, which was in the top five most abundant GRs in head appendage tissue, is an orthologue of DmelGr28bD. In *D. melanogaster*, DmelGr28bD is expressed in neurons of the labellum, legs, and cibarium but also in non-gustatory neurons of the abdomen and the arista segment of the antenna [82]. As such, DmelGr28bD has a nongustatory function regulating thermosensation by acting as a warmth sensor [83]. An orthologue of DmelGr28a, *Hirr_19652*, was also identified; as with other members of the DmelGr28 family, this gene is expressed in both gustatory and nongustatory tissues and was characterized as detecting RNA and ribonucleosides [84]. The ligands bound by GRs are not as defined as for ORs, and orthologues of *Drosophila* GRs with no known ligands were identified from the horn fly database, including DmelGr59f and DmelGr93a. These candidate gustatory receptors were detected at low levels in the horn fly, which is in keeping with their expression in subsets of taste sensilla and may also be due to low input from tarsi and wing margins in our study.

Benton et al. [64] classified the role of IRs in mediating *Drosophila* chemosensation. IRs are expressed in coeloconic sensilla of antennae, which are distinct from the sensilla types in which ORs are expressed. Fernandes and Pimenta [26] described coeloconic sensilla on the flagellum of the horn fly antenna. In addition to a role in olfaction, IRs are also present in labella, legs, wings, and the sacculus and arista of the antenna having a role in gustation, as well as thermosensation and hygrosensation (reviewed in [49,85]). IRs are a heteromeric complex comprising IR co-receptors and ligand-specific IRs that mediate the sensory response [86]. Phylogenetic comparison of the candidate horn fly IRs identified orthologues of Ir8a (*Hirr_11863*), Ir25a (*Hirr_4551*), Ir76b (*Hirr_4682*), and Ir93a (*Hirr_4050*), all of which function as IR co-receptors. As with the other receptor families, the candidate horn fly IRs are predominantly expressed in head appendages and this is reflected in the phylogenetic comparison, as the majority of the horn fly IRs are orthologous to *Drosophila* antennal IRs. These include DmelIr10a, 87a, 17b, 7c, 76d, 41a, 76a, 92a, 68a, 84a, 31a, and 75d (Figure 10). Although ligand specificities are still unknown for a number of these *Drosophila* IRs, *Hirr_9522* and *Hirr_7081* were orthologous to DmelIr40a and DmelIr21a that have a role in detecting moisture and thermal response [87,88], respectively, both of which are essential to horn fly oviposition [23]. The remaining horn fly candidate IRs are part of the *Drosophila* Ir20a clade that includes IRs expressed in neurons of taste organs, such as the labellum, legs, pharynx, and wing margin [89].

## 5. Conclusions

Integration of push–pull strategies in the management of livestock pests is a viable option for controlling pest fly populations [90]. The identification of natural product compounds that successfully repel horn fly adults and the evidence of repellent effectiveness in the field [8,9,10,11,12,13,14], while short-lived, supports the pursuit of this tool as an option for non-pesticide fly control. A further understanding of horn fly chemosensory pathways will provide additional targets for pest control development. Using an RNA-seq approach, we identified 153 chemosensory-related genes from the horn fly with a putative role in sensory perception, including carrier proteins and chemosensory receptors. There was no evidence for sex-specific and limited evidence for sex-biased expression of the chemosensory gene family members in this study, which may reflect the shared on-host microenvironment of females and males. Legs and wings harbor sensilla with a chemosensory function, and while these were included in the headless bodies dataset, further consideration will be made to resolve expression of the candidate horn fly chemosensory genes in these additional appendages. This dataset reflects one snapshot in time, and further studies of differences in temporal expression patterns of these candidate genes as they relate to reproductive and blood-fed state is warranted. Ultimately, functional characterization of these molecules will be essential to identifying those that are critical to horn fly behaviors, such as mating and oviposition.

## Figures and Tables

**Table 1:** Summary of transcriptome data from horn fly head appendages and headless bodies.

GenBank Accession (Sequence Read Archive)	Sample Dataset Pool	Total Number Read Pairs	Total Number Bases
SRR12763132	Female head appendage	19,605,733	4,901,433,250
SRR12763131	Female headless bodies	28,525,717	7,131,429,250
SRR12763130	Male head appendage	20,632,837	5,158,209,250
SRR12763129	Male headless bodies	51,464,662	12,866,165,500
For *de novo* transcriptome		120,228,949	30,057,237,250

**Table 2:** Differentially expressed candidate odorant binding protein (OBP) genes in fed, mated adult horn flies.

Sequence ID	Condition ^a^		Log_2_ Fold Change ^b^	
	A	B	A	B
*Hirr_10315*	fh > fb	mh > mb	8.04	8.21
*Hirr_11611*	fh > fb	mh > mb	3.95	3.64
*Hirr_14454*	fh > fb	mh > mb	12.98	14.15
*Hirr_1669*	fh > fb	mh > mb	6.99	3.15
*Hirr_18675*		mh > mb		2.38
*Hirr_2780*	fh > fb	mh > mb	7.67	7.86
*Hirr_4331*	fh > fb	mh > mb	11.84	12.01
*Hirr_4332*	fh > fb	mh > mb	5.43	3.99
*Hirr_5093*	fh > fb	mh > mb	3.82	3.87
*Hirr_5094*	fh > fb	mh > mb	9.55	9.09
*Hirr_5095*	fh > fb	mh > mb	8.73	7.18
*Hirr_5676*		mh > mb		2.6
*Hirr_577*	fh > fb	mh > mb	5.92	3.8
*Hirr_7363*	fh > fb	mh > mb	3.85	5.11
*Hirr_7975*	fh > fb		2.69	
*Hirr_8159*	fh > fb		2.06	
*Hirr_9170*	fh > fb	mh > mb	8.87	9.14
*Hirr_9619*	fh > fb	mh > mb	8.61	9.2
*Hirr_9620*	fh > fb	mh > mb	8.35	8.58
*Hirr_996*	fh > fb	mh > mb	9.19	9.6
*Hirr_10749*	fb > fh	mb > mh	2.1	3.02
*Hirr_1085*	fb > fh	mb > mh	5.06	2.61
*Hirr_12458*	fb > fh	mb > mh	3.04	6.25
*Hirr_223*	fb > fh	mb > mh	2.82	2.68
*Hirr_22727*	fb > fh	mb > mh	3.09	3.6
*Hirr_22826*		mb > mh		2.68
*Hirr_23217*	fb > fh	mb > mh	2.19	5.08
*Hirr_23217*	fh > mh		3.91	
*Hirr_326*	fb > fh	mb > mh	3.95	3.55

^a^: RNA-seq datasets from female head appendages (fh) and male head appendages (mh) were compared with those from female headless bodies (fb) and male headless bodies (mb) to identify differentially expressed transcripts; ^b^: Significant differences were based on an adjusted *p* < 0.01 calculated using the Benjamini–Hochberg false discovery rate (FDR) method with an FDR of 0.05.

## Supplementary Materials

The following are available online at https://www.mdpi.com/2075-4450/11/11/816/s1, Supplementary File 1: Annotated horn fly transcriptome representing genes expressed in head appendages and headless bodies of adult fed, mated horn flies; Supplementary File 2: Results of hierarchical clustering (fastcluster) and subsequent gene ontology analysis (GOSeq) of transcripts comprising each cluster; Supplementary File 3: GOSeq enriched categories of differentially expressed genes identified between the head appendage and headless bodies RNA-seq datasets; Supplementary File 4: Differentially expressed chemosensory gene family members; Figure S1: Amino acid sequence alignment of *H. irritans* Minus-C OBP genes.
