# Supplementary material for: Chemosensory-Related Gene Family Members of the Horn Fly, Haematobia irritans irritans (Diptera: Muscidae), Identified by Transcriptome Analysis
Source: Insects. 2020 Nov 19;11(11):816. doi: 10.3390/insects11110816 (PMC7699325; doi:10.3390/insects11110816)
Supplement: Supplementary file 1 [file insects-11-00816-s001.zip › Figure_S1.pdf]

```
                C1                                  C2  C3
ScalObp71     -----------MNK-----CAFIILLLWAFS-LCQVYAEILDLGKRPSWYPDDSPEI--- 40
Hirr_10749    -----------MKK-----FAYIIILLCWSCVMIREVYAEILDLGKRPSWYPDDGPEI--- 41
ScalObp70     --------MNRMKTLEQGFIIVIGI----------VLLTASNAAQRPDWFPENRAEI--- 39
Hirr_23522    -----------MKI-----FFYVFA----------MLIAVSMAFKRPDWYPKNGIEI--- 31
ScalObp68     -----------MKR-----LTIIFA----------FIVSASAVFERPDWYPENGVEI--- 31
ScalObp69     --------MRNMKQ-----LLVICA----------IILSASAAFERPDWYPENGSQI--- 34
Hirr_18958    ------------------------------------------------------------ 0
ScalObp67     ------MLISKMFN-----TIFAFL----------MLFGLGILAERPEWYPKNAAEI--- 36
Hirr_22727    -----------MNK-----IVFVLI----------VLSSSMIFAERPSWYPENSAEL--- 31
ScalObp64     -----------MTK-----LIIGLV----------LLALTMALAERPSWYPENAAEL--- 31
Hirr_15556    -----------MSK-----IIGVFL----------LLSITAILAERPSWYPENAAEL--- 31
Hirr_18389    LQINLQTLNSKMTK-----FIVVLL----------VLSMTMIFAERPEWYPENAAEL--- 42
Hirr_5709     --------------------------------------------HRPEWYPENSLDI--- 13
ScalObp65     -----------MHK------FTVLL----------IPVLSLVIAQRPDWLPENSPEI--- 30
ScalObp66     -----------MLK------LTVLL----------IPVLSLVIAQRPDWYPENSLDI--- 30
ScalObp74     -------------M-----FYILVL----------ILTARTILAQRPDWYPENPAKI--- 29
ScalObp72     -----------MNR-----LLVVFL----------ILFLSKTFAERPDWYPENAAEI--- 31
ScalObp73     -----------MIK-----LLIGCI----------MISITLILGERPEWYPRNASLF--- 31
ScalObp10     ------------MK-----FAAVVL-------------FALFAVASAEYKLRNQEDLMKA 30
DmelObp99c    -----------MLK-----YLIVAL-------------ALCAVAHADDWTPKTGEEIRNI 31
ScalObp62     ------------MK-----FMVILL-------------AIFACVSADDWTPKKSREIREI 30
ScalObp61     ------------MK-----YFVALL-------------VIVACVSAEEWKPKTADEIKTL 30
ScalObp63     --------MFEAIK-----YLIILL-------------AVVVCISADDWKPKVINELKSI 34
                C1                                  C2  C3
ScalObp71     EAECMEDFTISEESLKQIR-NLQIANTPAVRAYLLCFLKETNVYRPDKGPEIKRIAWSLK 99
Hirr_10749    EAECVEDHSISKETLDDIK-MLHIDNTPTVRAYLLCYLKETNVYRPEKGPELRRIAWSLK 100
ScalObp70     EANCLMENSIDDETLTNIR-QLKLDDTPQIRSLIMCSLINTNAYRPDREPEADRLVVAFQ 98
Hirr_23522    ENACKAKICPNEDILNKLR-KLQIEDNDKTRQLIMCCLKNTNVYRPGNGPEADRIGVAFE 90
ScalObp68     EALCSAKLADDDIILNKLR-KLEIEDNEKTRELILCCLKNTNVYREDHGPEADRIAVAFE 90
ScalObp69     EADCMSKIAVGDDILNKLR-KLQIDDNEKTRDLILCCLRNTNVYRPGQVPEADRIAVAFQ 93
Hirr_18958    --DCMAKISVSDSILQNLR-RLKIDDNEETRDLILCCLRNTNVYRPGYGPEADRIAVAFQ 57
ScalObp67     DAECMKKHVLSAEIMEKLKNDFTLEDTPTMRSLVFCSALGKQVFRPNVGFEAVRVVEGLQ 96
Hirr_22727    DAECMKKHPVSAEIIEKVR-SFHLEDTPNMRALVYCGATSKNVYIPGEGFNSERFVEGMK 90
ScalObp64     EVKCMKEHAVSPETVANMR-AFNLDEAPAIVAVLFCSGKAKKIYTPELGFVPERFAYAMK 90
Hirr_15556    EVNCMKEHSVSPETVSKMR-GFHLEEAPAIVSVLFCSGKAKNIYTADLGFVPERFAYGMK 90
Hirr_18389    EVNCMKDHNVTPEIVAKIR-SFHLDDTPEIRDVVYCSAKAKNIYRPGVGFEPERFVLGVR 101
Hirr_5709     EAECLRKNGLDYETMNDIL-SLRVEDTPALRSFLYCSAVGKNVYRPDIGFEMDRFELGLK 72
ScalObp65     EAECLAKNCLSPEIMNDIL-NLHVEDTPAMRAFLFCAAVRKNVYRPEIGFEIDRFDMGLK 89
ScalObp66     EAECMAINCLSPEIMYDIL-NLHVEDTPAMRAFLFCAAVGKNVYRPEIGFEIDRFDMGLK 89
ScalObp74     EEQCMREHSITPEIWSTIR-SFHLDDTPNVGSFFLCLNTKKGVFRPEKGFEPERLAIGIR 88
ScalObp72     EADCKKENGITPEIWSQML-SLDLEDTPPVRSVLLCLVKRKTVYRPENGFEAERLQVGLQ 90
ScalObp73     EKACLVENDLRPDQWARMR-TMHLEDTPAERAFLLCLVKTKNVFRPEKGFEAERLRLGLQ 90
ScalObp10     RKECMEAKKVTPELIEKYK-KFDFPDDETTRCYIECVFEKFELFDAKEGFKNDNLVAQLG 89
DmelObp99c    RVECLKENPLSNDQISQLK-NLIFPNEPDVRQYLTCSATKLGIFCDQQGYHADRLAKQFK 90
ScalObp62     RQACMETISLTESQKKKIE-NFDFPNDEAVRKYIVCTLEKMEICCTSHGFHVNRLIKQTN 89
ScalObp61     RAACLQEHPLSEEQMNRMK-VFDFPDEEAVRKYLMCTSEKMDVYCPHEGYHADRIAKQFK 89
ScalObp63     RDECLNEVPVTEDQRKRMN-ALDFPNEEAVRNFILCAVQKLGIYSTEHGYYADRLTKQFN 93
                 *           C4     :  . :C5     . *C6               ..
ScalObp71     QNYHLNKCDADMIRDCIEEQGDQ-DLKDYAFFNIIKCVFHKNRIYGTNKLSPIYL----- 153
Hirr_10749    EYLKLNKCDADMIRDCIEEHGAQ-DLKDYAYFHIIKCAYEKAPDRCLAKVKDN*------ 152
ScalObp70     QNLKRD-CDIDMVESCIDQYKHL-QPDDYKYFTIIKCIFNEAPTKCTLIK---------- 146
Hirr_23522    QSLRLH-CEADLIAGCIQTFKDT-TNEYEQFFNVIKCIYDVAPEKCKPE*---------- 137
ScalObp68     QSLKLH-CEPELIQECSQKFKEE-KPESYEFFRIIKCIYDEAPQKCKP------------ 136
ScalObp69     QSLKLN-CEEDLINGCIPKYKDE-KPENYQFFRIIKCIYDEAPARCKP------------ 139
Hirr_18958    QSLKLN-CDIDLIQGCIPKFIHE-MPEKYQFFRTIKCIYDEAPQRCLP*----------- 103
ScalObp67     ENAKLN-CSLEAVQHCADQFRNA-NSNEAMFFSTMKCLFDDISENCSKMKETE------- 147
Hirr_22727    ESSKMN-CNMDSVRNCADKHKNA-ESDEAIFFNVLKCVFDDINGICNKVV*--------- 138
ScalObp64     TNVKMD-CNVDYIRNCAEQHKDV-QPVDTMYFKVVKCVFDNREGHCTKV----------- 137
Hirr_15556    QNVKLD-CDVDFIRNCAETHKEV-QPAETMYFRVVKCVFDNREGHCKKVE*--------- 138
Hirr_18389    ESVKMD-CNINYIRSCGEKHKYV-QPE--------------------------------- 126
Hirr_5709     YKIKLD-CNKEFLNSCGELYKEE-ISPESLYFHIVKCVFDNSEDNCIRTH*--------- 120
ScalObp65     YKIKID-CSKEFLNYCVELYQSE-ENPESLYFYIVKYVSNTTTEVP-------------- 133
ScalObp66     YKIKID-CSKEFLNYCVEVYQWE-ENPESFYFYIVKCVFDNAEDYCIRIP---------- 137
ScalObp74     MTTKVD-CDVNMIRNCGDRYKEL-KPHDHMILNIIKCIFENKEGNCRKIQ---------- 136
ScalObp72     QVAKRK-CDLNHIKDCGEKFCDL-EPEDFKIFSIFKCTLDDRHEKCEKVE---------- 138
ScalObp73     QSIKAD-CDIEHIEHCKQMYGHL-KPDDVMVFQIAKCVCDVKDEKCRKLIKNEL------ 142
ScalObp10     QGKDNKDAVKTDVEKCADKNEQK-SDSCSWAFRGFKCFIKSNLPLVMDSLKKN------- 141
DmelObp99c    MDLSEE-EALQIAQSCVDDNAQK-SPTDVWAFRGHQCMMASKIGDKVRAFVKAKAEEAKK 148
ScalObp62     KDLGSE-QGKDVVEECLRNNPKGDNEIEIYAHDVYTCLIDEVTKIIES------------ 136
ScalObp61     MDMVED-DVKKLAEDCISSNPKGDKANDVHVYEVHKCLMASEVGQKVKSYIKSRQEQLAK 148
ScalObp63     SELHGD-DGKEPVDNCLNNNPQGYKENDVYVYEMHLCLIEARKEHAKRHK-----EQ--- 144
                     .        *
```

Figure S1A. Amino acid sequence alignment of horn fly candidate Minus-C OBPs with a Minus-C OBP from *Drosophila* (DmelObp99c) and *Stomoxys calcitrans* (Scal). ScalOBP10, a classical OBP, is included in the alignment for reference to location of conserved cysteine residues. Conserved cysteine residues shared between sequences are shaded in blue. The C_2 and C_5 cysteines that are missing from the Minus-C OBPs are shaded in orange and within these the cysteine in ScalOBP10 is in bold text. The cysteine shaded in pink reflects the alternate cysteine residue located seven residues upstream of C_4.

```
                                                                     C1
ScalObp10       -MKFAAVVLFA----LFAVAS----AEYKLRNQEDLMKARKECMEAKKVTPELIEKYKKF 51
DmelObp99a      -MK-VFVAICV----LIGLAS----ADYVVKNRHDMLVFRDECVKELAVPVDLVEKYQKW 50
DmelObp99d      ------MIHLRLAIIC---WSCLLIVMATTTEAASVW--------KLPTAEMVYEDLEKC 43
DmelObp8a       MMRRSQIWLLRLLLLLVEMTPPAIPVPMRSSQSLALLRARDQCGRELTAAQRL--QLDRM 58
MdomObp58       -MKSRTFVAL----LLCNIL---ILVTGQNTISDNFYDKSEKCFDQLHVPQRYKATFQAF 52
Hirr_326        -MKSFAFCG-----LFLNIW---LSVHGQNTISDRFFDVSEKCFEQLHVPQRYKSEFQQF 51
ScalObp58       -MKLVAFIL-----LFLS-L---PAVFGQNTISDQFFSKSEICFELLHVPNRYRSEFGNF 50
                      .                  .         .            .

                         C2  C3                                        C4
ScalObp10       DFPDDETTRCYIECVFEKFELFDAKEGFKNDNLVAQL-GQGKDNKDAVKTDVEKCADKNE 110
DmelObp99a      EYPNDAKTQCYIKCVFTKWGLFDVQSGFNVENIHQQLVGNHADHNEAFHASLAACVDKNE 110
DmelObp99d      RQESQEEDAATLRCLVKKLGLWTDESGYNARRIAKIFAGH--NQMEELMLVVEHCNRKEQ 101
DmelObp8a       QFEDAAHVRHYLHCFWSRLQLWLDETGFQAQRIVQSFGGERRLNVEQALPAINGCNAKTS 118
MdomObp58       RYPDEEIVHKYVHCLAMKLEIWTNRSGFNIEKIYNQYRN--RVNDEIMLPTISNCNRSAQ 110
Hirr_326        RYADTEMVHKYIHCVSTELHIWNNRSGFDIEKIYNEYHA--RANDEVMLPIISNCNRTTQ 109
ScalObp58       SYADEEVVHKYIHCVTTELDIWNDRSGFNIEKIYHQYRN--RANDEVMLPIISNCNRSSQ 108
                   .       :.*.  .  ::  . *:.  .:          : :     :  *  . .

                         C5      C6
ScalObp10       Q----KSDSCSWAFRGFKCFIKSNLPLVMDSLKKN-------------    141
DmelObp99a      Q----GSNACEWAYRGATCLLKENLAQIQKSLAPKA------------    142
DmelObp99d      D----TSHLDDWAFLAYRCATSGQFGHWVKDFMSQKDVDQ--------    137
DmelObp8a       SRGSGTQTVVDWCFRAFVCVLATPVGEWYKRHMSDVINGNA-------    159
MdomObp58       N-----SNKELWCYRAFLCILNTDVGKWFKEDVQRSRQANNVPNGHH-    152
Hirr_326        N-----SNKELWCYRAFVCILNTDVGKWFKEDVQRSRQAG-IPNGYH*    150
ScalObp58       N-----NNKELWCYRAFVCILNTDVGQWFKEDVQRKRQAN-ISNGHH-    149
```

Figure S1B. Amino acid sequence alignment of horn fly candidate Minus-C OBP, *Hirr_326*, with Minus-C OBPs from *Drosophila* (DmelObp99a, 99d) and *Stomoxys calcitrans* (Scal). ScalOBP10, a classical OBP, is included in the alignment for reference to location of conserved cysteine residues. Conserved cysteine residues shared between sequences are shaded in blue. The C_2 and C_5 cysteines that are missing from *Hirr_326* are shaded in orange and within these the cysteine in ScalOBP10 is in bold text. The cysteine shaded in pink reflects the alternate cysteine residue located three residues downstream of C_5.
